# Individual differences in the physiological effects of forest therapy based on Type A and Type B behavior patterns

**DOI:** 10.1186/1880-6805-32-14

**Published:** 2013-10-02

**Authors:** Chorong Song, Harumi Ikei, Juyoung Lee, Bum-Jin Park, Takahide Kagawa, Yoshifumi Miyazaki

**Affiliations:** 1 Center for Environment, Health and Field Science, Chiba University, Chiba, Japan; 2 Korea Forest Service, Daejeon, Korea; 3 Depart of Environment & Forest Resources, Chungnam National University, Daejeon, Korea; 4 Forestry and Forest Products Research Institute, Ibaraki, Japan

**Keywords:** Forest bathing, Urban environment, Pulse rate, Blood pressure, Individual difference, Type A behavior pattern, Type B behavior pattern, Kwansei Gakuin (KG) daily life questionnaire

## Abstract

**Background:**

In recent years, the physiological relaxation effects of natural environments have been widely exploited, and although individual differences in the effects of forest therapy are known, assessment methods have not been clearly established. This study used a classification based on Type A and Type B behavior patterns to explain individual differences in physiological responses to forest environments.

**Methods:**

We performed physiological experiments in 44 forest and urban (controls) areas. In total, 485 male university students (age, 21.8 ± 1.6 years) participated in the study. The subjects were asked to visit forest or urban environments randomly and observe each landscape for 15 min. The subjects’ pulse rates and blood pressures were tested to evaluate their physiological responses. The Kwansei Gakuin daily life questionnaire was used to identify Type A and Type B behavior patterns in subjects.

**Results:**

The pulse rate was significantly lower in the Type B group after exposure to forest areas than after exposure to urban areas, whereas no significant difference was observed in the Type A group. In addition, the pulse rate was significantly lower in the low scoring subjects in the Type B group, which was consistent with changes in their diastolic blood pressure.

**Conclusions:**

These results suggest that individual differences in pulse rate and blood pressure in response to forest environments can be explained by Type A and Type B behavior patterns.

## Background

It is widely believed that contact with the natural environment can improve the physical and mental health of people. Since 1980, many studies have demonstrated a significantly positive relationship between people’s exposure to natural environments and their health. Several questionnaire-based studies have reported restorative effects related to psychological stressors or mental fatigue [1-3] and improved mood states and cognitive function [4-7]. Improved physiological measurement techniques in recent years have generated further scientific evidence based on physiological parameters. Studies on the physiological effects of relaxation in forest environments have tested such parameters as cerebral activity in the prefrontal cortex [8], pulse rate [9-13], blood pressure [10,11,13,14], heart rate variability [9,10,12-14], salivary cortisol concentration [8,9,11-13], and natural killer cell activity [15-18]. A significant amount of scientific evidence has been reported [19-21]. Park *et al*. [20] reported that forest therapy mitigated stress and led to biological relaxation based on the results of experiments using 420 subjects in 35 locations throughout Japan. Forest therapy is a health-promotion method and uses medically proven effects of forests, such as relaxation, that can improve the health of the body and mind. It is aimed at achieving a preventive medical effect by improving the immune system response.

However, individual differences within these effects have been noted, and this phenomenon has posed several questions in a variety of fields that must be clarified. Thus far, methods for interpreting these individual differences have not been established. Previous studies on individual differences in the generated responses have produced distinctive results for individuals with Type A and Type B behavior patterns. Type A and Type B behavior patterns were proposed by Friedman and Rosenman [22] on the basis of specific behavior patterns in patients with heart disease. The Type A behavior pattern can be defined as an overt behavioral syndrome related to lifestyle that is characterized by excessive competitiveness, striving for achievement, aggressiveness, time urgency, acceleration of common activities, restlessness, hostility, hyperalertness, explosiveness of speech amplitude, facial musculature tension, feelings of struggling against the limitations of time, and insensitivity to the environment [23]. Type B behavior pattern is characterized by the absence of Type A characteristics. Miyazaki and Tsunetsugu [24] examined individual differences in hemoglobin concentration in the prefrontal cortex when chocolate was used as a gustatory stimulus. The activities of the prefrontal cortex stimulated by chocolate significantly increased in the Type B group, whereas the Type A group showed no changes in their activities. The results showed that physiological responses in Type B and Type A subjects were significantly different. Park *et al*. [25] investigated changes in cortisol concentration after administering a eucalyptus-flavored drink. The concentration of salivary cortisol significantly decreased after ingestion of a eucalyptus-flavored drink in the Type B group, but changes in the Type A group were statistically insignificant. Furthermore, Lee and Watanuki [26] reported differences in the cardiovascular responses of Type A and Type B subjects after visual stimulation using displeasure-evoking images and nature video clips. However, the majority of the previous studies were laboratory experiments, which were limited by small sample sizes. It is necessary to use both laboratory experiments and field experiments with large sample sizes to assess individual differences.

Therefore, a field experimental design was used to study individual differences in the physiological effects of forest therapy based on Type A and Type B behavior patterns in a sample size of 485 subjects. Furthermore, because this study used a large sample size, we examined additional details from the classification of the Type A and Type B groups.

## Methods

### Subjects and study sites

**Figure 1:**
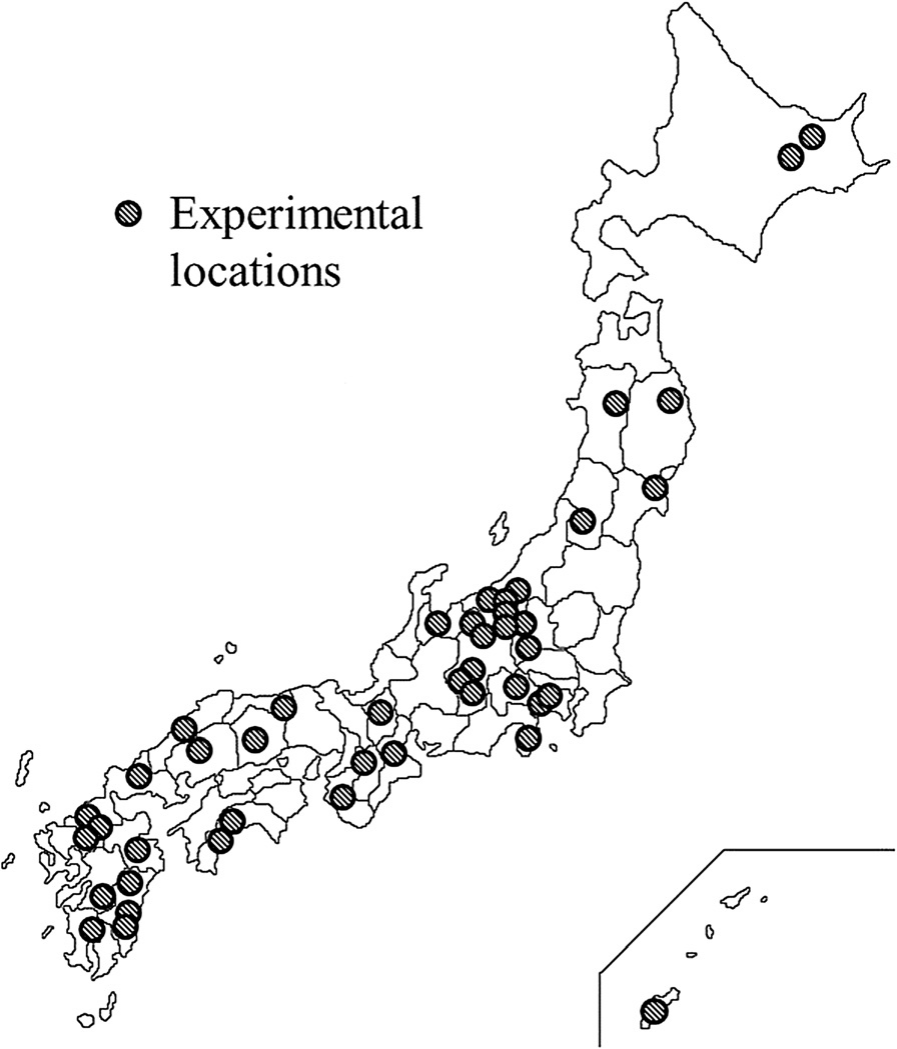
Map showing experimental locations.

From 2005 to 2010, we performed physiological experiments in 44 areas of Japan (Figure 1). Two study sites, a forest and an urban environment, were selected for each of the 44 areas. Twelve male university students participated in each of the experiments (in total, 528 subjects), and no student reported a history of physical or psychiatric disorder. Of these, we were able to measure the blood pressure and pulse rate of 485 subjects (21.8 ± 1.6 years old) for analysis. The study was performed according to the regulations of the Ethics Committee of the Center for Environment, Health, and Field Sciences, Chiba University or the Institutional Ethics Committee of the Forestry and Forest Products Research Institute in Japan.

### Measurements

A digital blood pressure monitor based on oscillometric methods (HEM1000, Omron, Japan) was used to measure the pulse rate and blood pressure (systolic and diastolic blood pressure) in the right upper arm.

We used the KG daily life questionnaire to determine Type A and Type B behavior patterns [27]. The KG daily life questionnaire comprises 55 items (44 that determine Type A and Type B behavior patterns and 11 irrelevant or dummy questions). The subjects were asked to choose one of the three options as a reply (yes, ?, or no) for each question; with respective scores of 2, 1, or 0 for the replies. It was found that a score of <43 points indicated a Type B behavior pattern, while >44 points indicated a Type A behavior pattern.

### Experimental design

On the day before the experiment, the subjects were fully informed about the aims and procedures involved. After receiving a description of the experiment, the subjects signed an agreement to participate. All subjects were instructed to reside in a hotel with identical single rooms and eat the same category of food during the ongoing procedure, to minimize individual differences caused by these factors. Alcohol and tobacco were prohibited, and caffeine consumption was controlled. After orientation, the subjects visited and previewed the forest and urban study sites before commencement of the experiment. Measurement practice of the physiological indices was performed at the hotel. The subjects were asked to complete the KG daily life questionnaire in their individual rooms after dinner.

**Figure 2:**
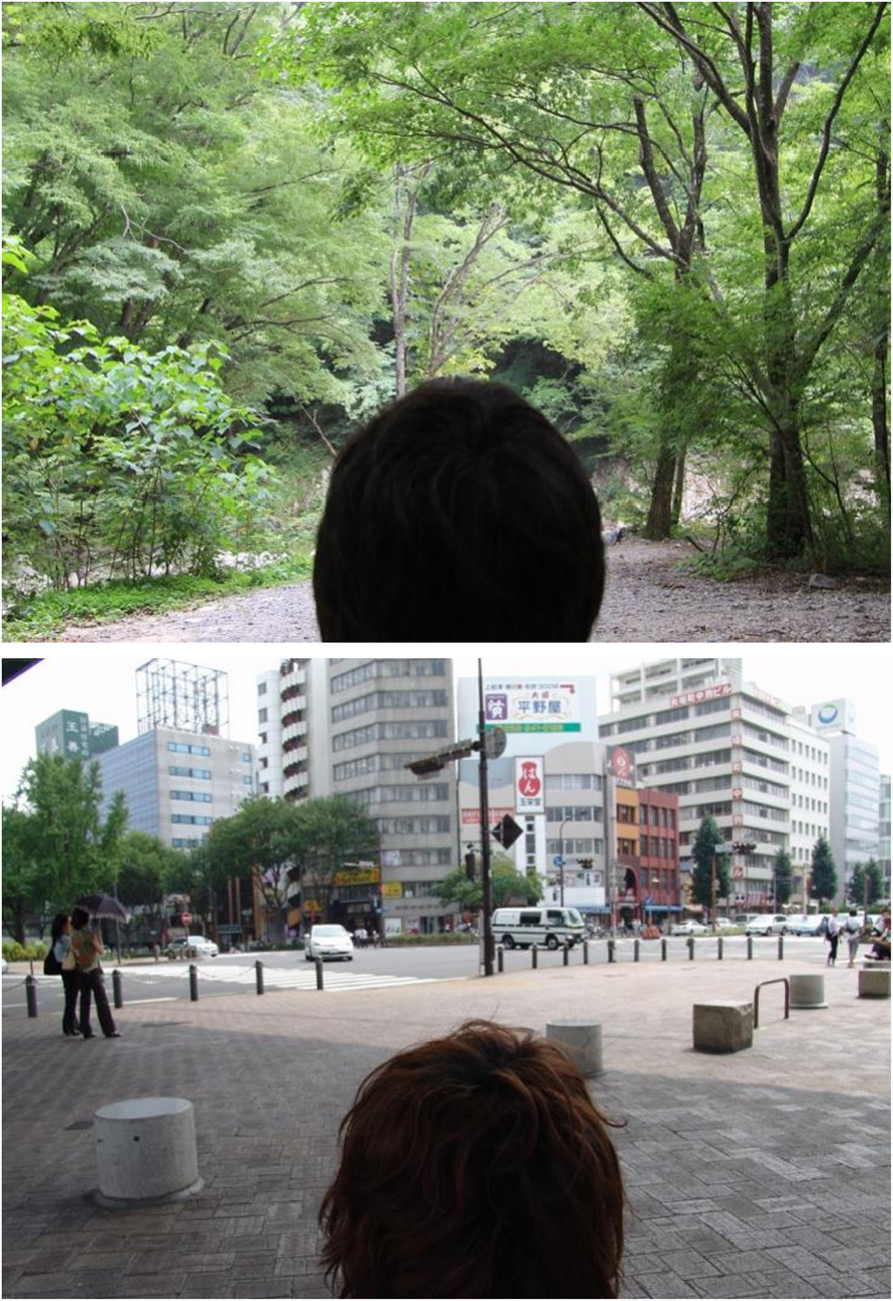
**An example of experimental landscapes in the experimental area.** Top: Forest area, Bottom: Urban area.

Twelve subjects were randomly assigned to the forest or urban environment groups for each of the 44 areas, to eliminate the ordering effect. On the first day of the experiments, six subjects were sent to a forest site, and the other six subjects were sent to an urban site. On the second day, the sites were interchanged. After arriving at the site, each subject was asked to sit on a chair and view the landscape (the forest or urban environment) for a period of 15 min in the afternoon. Figure 2 shows examples of two experimental viewing areas. Physiological measurements were recorded before and after viewing. We measured each physiological index three times; mean values were used for comparative purposes.

### Data analysis

We used the differences in the physiological values, i.e., 'postviewing’ minus 'previewing’, to determine the change in the physiological response to viewing. The response data from forest and urban environments were compared between Type B and Type A subject groups and between four subgroups, which were obtained by dividing each of the Type A and Type B subject groups according to their KG daily life questionnaire scores. We divided each group according to the median. The below-median Type B subgroup (*N* = 252) was the low scoring Type B subgroup (*N* = 126), and the above-median Type B subgroup was the high scoring Type B subgroup (*N* = 126). The Type A group was subdivided in the same way.

A two-way repeated measures analysis of variance (ANOVA) with one between-subjects factor (that is, Type A or B behavior pattern with two or four levels) and one within-subjects factor (that is, environment with two levels) was performed, to determine whether the individual responses to the two environments differed significantly with respect to the Type A and Type B behavior patterns. Furthermore, a simple effect test with Bonferroni correction was performed for the post-hoc analysis. SPSS 20.0 (SPSS Inc., Chicago, IL, USA) was used to perform statistical analysis of the physiological data. In all cases, values of *P* < 0.05 were considered to indicate statistical significance.

## Results

**Figure 3:**
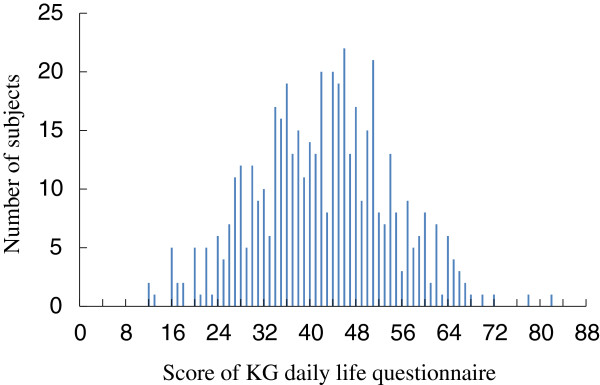
**Distribution of the Kwansei Gakuin (KG) daily life questionnaire scores.***N* = 485.

**Figure 4:**
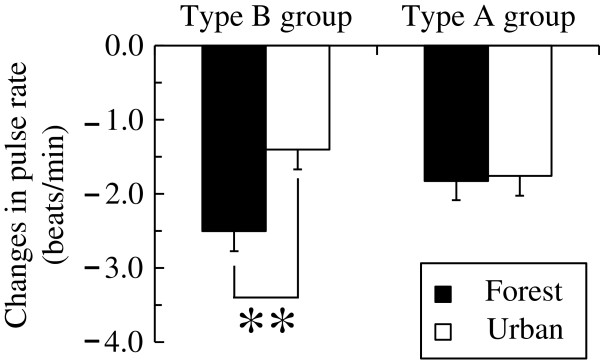
**Changes in pulse rates in the Type B and Type A groups after exposure to the two environments.** Type B group: *N* = 252, Type A group: *N* = 233. Mean ± standard error. **P < 0.01 by two-way analysis of variance (ANOVA) for repeated measures with post-hoc contrasts.

The distribution of the KG questionnaire scores is shown in Figure 3. Based on the scores in the KG daily life questionnaire, we divided the subjects into Type B group (12 to 43 points, *N* = 252) or Type A group (44 to 82 points, *N* = 233). Figure 4 shows a comparison of the changes in the pulse rates of Type B and Type A subjects. The ANOVA showed a significant main effect for the environment [*F* (1,483) = 5.720, *P* < 0.05] and environment × the behavior pattern interaction [*F* (1,483) = 4.464, *P* < 0.05], which indicated that the pulse rates were changed by the environment and that the differences in the pulse rates for the forest environment and urban environment depended on the subject’s behavior type (i.e., Type A or Type B behavior pattern). The simple main effect of the difference between the two environments was significant in the Type B subject group (*P* < 0.01). For this group, viewing a forest environment decreased the pulse rate by 2.5 beats/min (from 65.7 beats/min to 63.2 beats/min); this was significantly lower than the change after viewing an urban environment. However, the results for Type A subjects exhibited no significant differences between the forest and urban environments.

**Figure 5:**
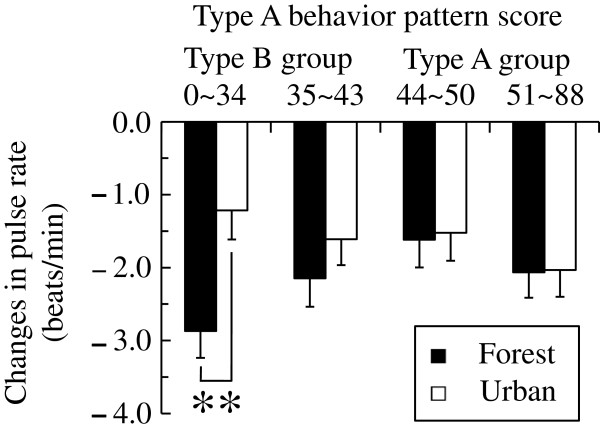
**Changes in the pulse rates of the four subgroups after exposure to the two environments.** Low scoring Type B subgroup: *N* = 126, high scoring Type B subgroup: N = 126, low scoring Type A subgroup: *N* = 116, high scoring Type A subgroup: *N* = 117. Mean ± standard error. **P < 0.01 by two-way analysis of variance (ANOVA) for repeated measures with post-hoc contrasts.

In addition, we analyzed four subgroups, which were obtained by dividing each of the two Type A and Type B subject groups on the basis of the scores of the KG daily life questionnaire. Figure 5 shows a comparison of changes in the pulse rates of the four subgroups. The ANOVA detected a significant main effect for the environment [*F* (1,481) = 5.428, *P* < 0.05] and a trend in the environment × the behavior pattern interaction [*F* (3,481) = 2.121, *P* = 0.097], which indicated that the pulse rates for the environment and forest and urban environments varied among the four subgroups. The simple main effect of the difference between the two environments was significant in the low scoring Type B subgroup (12 to 35 points, *N* = 126, *P* < 0.01). The low scoring Type B subgroup exhibited a significant reduction of 1.7 beats/min after the subjects were exposed to the forest area compared with the pulse rate of the subjects after exposure to urban areas. In the other three subgroups (high scoring Type B subjects: 35 to 43 points, *N* = 126; low scoring Type A subjects: 44 to 50 points, *N* = 116; and high scoring Type A subjects: 51 to 82 points, *N* = 117), differences in the pulse rates on exposure to the forest and urban areas were statistically insignificant.

**Figure 6:**
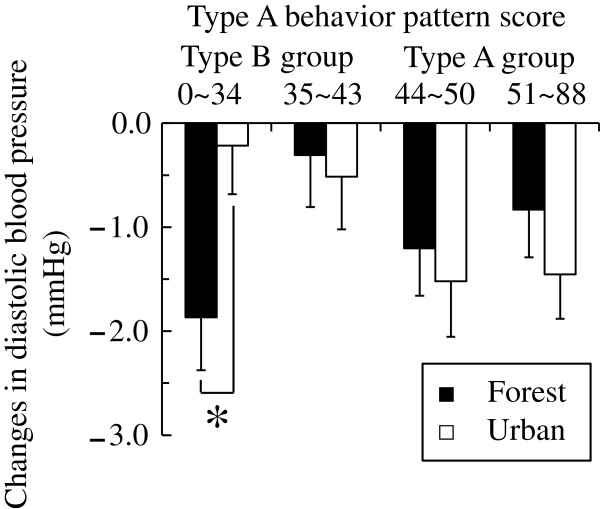
**Changes in diastolic blood pressure of the four subgroups after exposure to the two environments.** Low scoring Type B group: *N* = 121, high scoring Type B group: *N* = 122, low scoring Type A subgroup: *N* = 116, high scoring Type A subgroup: *N* = 116. Mean ± standard error. **P < 0.01 by two-way analysis of variance (ANOVA) for repeated measures with post-hoc contrasts.

Analysis of diastolic blood pressure showed no significant difference between the forest and urban environments compared with: the Type B subject group (12 to 43 points, *N* = 243) and Type A subject group (44 to 82 points, *N* = 232); however, ANOVA of the four subgroups, which were obtained by dividing each of the two Type A and Type B subject groups on the basis of the scores in the KG daily life questionnaire, showed a trend in the environment × the behavior pattern interaction [*F* (3,471) = 2.457, *P* = 0.062], which suggested that there were differences in the diastolic blood pressure for the forest environment and urban environments in the four subgroups (Figure 6). The simple main effect of the difference in the diastolic blood pressure between the two environments was significant in the low scoring Type B subgroup (12 to 34 points, *N* = 121, *P* < 0.05). The low scoring Type B subgroup exhibited a more significant reduction for forest environments than for urban environments. In the other three subgroups (high scoring Type B subjects: 35 to 43 points, *N* = 122; low scoring Type A subjects: 44 to 50 points, *N* = 116; and high scoring Type A subjects: 51 to 82 points, *N* = 116), there was no significant difference in the forest and urban environments.

## Discussion

In this study, we aimed to elucidate individual differences in the physiological response to forest therapy by evaluating changes in the pulse rates and blood pressures in 485 subjects after forest therapy in field experiments, in which individuals were classified as having a Type A or Type B behavior pattern. The results showed that there was a significant reduction in the pulse rate after viewing forest scenes compared with that found after viewing urban areas, and that the reduction was related to the subject’s behavior pattern.

The reductions in the pulse rate and blood pressure after viewing the forest for 15 min confirmed that forest therapy induced a relaxed state. Although we only used blood pressure and pulse rate data in this study to examine the individual difference, previous studies that have used various physiological indexes have been reported. According to previous studies, staying in a forest also suppressed sympathetic nervous activity [10,12-14], increased parasympathetic nervous activity [9,10,12-14], decreased cortisol level [8,9,11-13], and reduced cerebral blood flow in the prefrontal cortex [8]. It has been suggested that human beings are more relaxed in forest environments. Thus, we considered that the reduced pulse rate and blood pressure in the present study reflected a relaxed physiological state. Classification into Type A and Type B subjects showed that the pulse rate reduction was significant in the Type B group; however, there was no significant difference in the Type A group. Thus, the differences in the physiological effects of forest therapy were only present in the subjects with the Type B behavior pattern. When all of the subjects were divided into four subgroups, there was only a significant decrease in the low scoring group of Type B subjects.

The Type A behavior pattern can be defined as an overt behavioral syndrome related to lifestyle, which is characterized by excessive competitiveness, striving for achievement, aggressiveness, time urgency, restlessness, hostility, feelings of struggling against the limitations of time, and insensitivity to the environment [23]. The Type A behavior pattern is also known to be linked to patients with heart disease. Therefore, previous studies of the Type A behavior pattern have primarily investigated increases in stress-related conditions related to sympathetic activity in Type A individuals. In general, the heart rate and systolic blood pressure of Type A individuals increased more than those of Type B individuals under stressful conditions [28], and muscular vasodilation and secretion of norepinephrine, epinephrine, and cortisol were more pronounced in Type A individuals [29,30]. Dembroski *et al*. [31] also reported that stressed Type A individuals exhibited a higher response in their sympathetic activity than Type B individuals, and Oishi *et al*. [32] suggested that stressors do not affect the parasympathetic activity in Type A individuals.

We observed that the pulse rate and blood pressure were significantly reduced in the Type B group after forest therapy but that there were no differences in the Type A group. This result is consistent with the findings of previous studies that investigated the Type A behavior pattern response to natural stimulation. Lee *et al*. [33] examined the correlation between the Type A behavior pattern and changes in systolic blood pressure after walking in a forest environment for 14 min. The systolic blood pressure was reduced in the Type B group, whereas it increased in the Type A group. Park *et al*. [25] investigated changes in the cortisol concentration in response to a eucalyptus-flavored drink. The cortisol levels were significantly reduced in the Type B group, whereas there were no changes in the Type A group. Miyazaki and Tsunetsugu [24] investigated the response of hemoglobin concentration changes in the prefrontal cortex to gustatory chocolate stimuli. There was a significant change in the Type B group, but no changes were observed in the Type A group. These studies also revealed that Type B subjects exhibited a more profound change in response to exposure to natural stimuli than Type A subjects, which agreed with our findings. However, the reasons for a higher response in the Type B group, particularly in the low scoring subgroup of Type B subjects, are currently unknown. Several previous studies have detected an increase in the sympathetic activity of Type A individuals under stressed states. However, forest therapy aimed to enhance parasympathetic activity [9,10,12-14,17]; thus, our results may require a different explanation from that given in previous studies. A possible future research area would be to investigate the mechanism of the physiological reaction in Type A and Type B individuals.

This study used forest therapy to evaluate the effects of physiological relaxation in a large sample of 485 subjects and detected individual differences in the relaxation effects, particularly in the subjects who exhibited a Type B behavior pattern. We suggest that the results of this study may help to elucidate physiological polymorphism, which is an important concept in physiological anthropology. However, this study was limited to men in their 20s; thus, verification of our results using subjects with different attributes is necessary.

## Conclusions

The pulse rate was significantly lowered by forest therapy, and the level of the reduction varied depending on whether the subjects exhibited a Type A or Type B behavior pattern. Individual differences related to changes in pulse rate and diastolic blood pressure after forest therapy were related to the Type A and Type B behavior patterns.

## Abbreviations

ANOVA: Analysis of variance; KG: Kwansei Gakuin.

## Competing interests

The authors declare that they have no competing interests.

## Authors’ contributions

CS carried out data acquisition, statistical analysis, interpretation of the results, and manuscript preparation. HI, JL, and BP were involved with the acquisition and interpretation of data. TK designed the study and was involved with data acquisition. YM had an important a role in the overall performance of this research, particularly experimental design, data acquisition, and manuscript preparation. All authors contributed to the preparation and are responsible for the final editing and approval of the manuscript.
